# Sound comparison of seven TMS coils at matched stimulation strength

**DOI:** 10.1016/j.brs.2020.03.004

**Published:** 2020-03-12

**Authors:** Lari M. Koponen, Stefan M. Goetz, Debara L. Tucci, Angel V. Peterchev

**Affiliations:** a Department of Psychiatry & Behavioral Sciences, Duke University, Durham, NC, 27710, USA; b Department of Electrical & Computer Engineering, Duke University, Durham, NC, 27708, USA; c Department of Neurosurgery, Duke University, Durham, NC, 27710, USA; d Department of Head and Neck Surgery & Communication Sciences, Duke University, Durham, NC, 27710, USA; e Department of Biomedical Engineering, Duke University, Durham, NC, 27708, USA

**Keywords:** Transcranial magnetic stimulation, TMS, Coil click, Sound, Stimulation strength, Hearing safety

## Abstract

**Background:**

Accurate data on the sound emitted by transcranial magnetic stimulation (TMS) coils is lacking.

**Methods:**

We recorded the sound waveforms of seven coils with high bandwidth. We estimated the neural stimulation strength by measuring the induced electric field and applying a strengtheduration model to account for different waveforms.

**Results:**

Across coils, at maximum stimulator output and 25 cm distance, the sound pressure level (SPL) was 98–125 dB(Z) per pulse and 76–98 dB(A) for a 20 Hz pulse train. At 5 cm distance, these values were estimated to increase to 112–139 dB(Z) and 90–112 dB(A), respectively.

**Conclusions:**

The coils’ airborne sound can exceed some exposure limits for TMS subjects and, in some cases, for operators. These findings are consistent with the current TMS safety guidelines that recommend the use of hearing protection.

## Introduction

Transcranial magnetic stimulation (TMS) activates cortical neurons by electromagnetically inducing an electric field (E-field) pulse with a coil placed on the subject’s scalp. In addition to the desired E-field stimulation, the pulse causes mechanical vibrations in the coil, producing a brief but very loud sound impulse—often referred to as TMS coil click [1,2].

The sound pulse can potentially cause hearing loss [3,4]. This risk can be mitigated with adequate hearing protection [5–7]. However, should the hearing protection fail, TMS can cause permanent hearing loss [3]. The sound also evokes unwanted neural activation of the peripheral and central auditory pathways [8–10]. Thus, the sound negatively impacts the specificity of TMS and confounds functional brain imaging data, which can be only partially mitigated with noise masking [11].

Due to these side effects, the TMS safety consensus group suggested in 2009 that “the acoustic output of newly developed coils should be evaluated and hearing safety studies should be conducted as indicated by these measures” [4]. Since then, specific stimulatorecoil combinations have been evaluated with hearing safety measurements [6,7], and a comparison between coils has been published [2,12]. We extend this previous work by quantifying the impulse characteristics of the airborne TMS sound and comparing the measurements of seven coils at normalized stimulation strengths. Moreover, we interpret the results for single pulses as well as for repetitive TMS (rTMS) in the context of various standards for sound exposure limits.

## Material and methods

### TMS devices

We tested three stimulators with a total of seven different figure-of-eight coils. The model information of the tested stimulators and coils is provided in Table 1. The “stimulator output” refers to the stimulator capacitor voltage, expressed as a percentage of its maximum (maximum stimulator output, MSO), which determines the TMS pulse amplitude.

### Sound recording

The sound associated with a TMS pulse is impulsive and resembles a small explosion, such as shooting a firearm, in terms of its duration and peak sound pressure [13]. Consequently, measurement protocols and safety regulations for such sounds provide a good reference for TMS noise characterization. Therefore, our measurement setup mostly followed the impulsive sound measurement procedures suggested in US Department of Defense standard MIL STD 1474E [14]. The duration of the TMS coil click (<20 ms) is brief compared to the interstimulus interval (>20 ms) of conventional rTMS pulse trains. Consequently, the sound of arbitrary TMS pulse trains can be synthesized from sound waveforms of a single TMS pulse. Thus, we only measured single-pulse TMS sounds.

A microphone was pointed at the center of the head-side of each coil at a distance of 25 cm (see Supplementary Fig. S1B). The 25 cm recording distance was selected to avoid the confounds of spatial fluctuations in the sound near-field [2,15] and to allow removal of the TMS electromagnetic artifact induced into the microphone hardware. The latter was possible since no sound from the coil could arrive at the microphone before about 730 μs—after the end of the TMS electromagnetic pulse. The ears of TMS subjects are much closer to the coil than 25 cm; for example, the shortest coil-to-ear distance is typically around 5 cm for primary motor cortex stimulation. The sound pressure level (SPL) from TMS coils decays approximately inversely with distance down to 5 cm [16] (see Table 2); therefore, the sound pressure at 5 cm from a coil is about 14 dB higher than the sound pressure measured at 25 cm. Such distance-adjusted numbers are provided in parentheses in the results. Similarly, the ears of the TMS operator are usually slightly farther: 30–50 cm from the coil is typical, based on measuring the normal pose of three TMS operators in our lab, resulting in a 2–6 dB lower SPL.

We sampled 10 pulses per stimulator output at 10%–100% of MSO in 10%-MSO steps. The pulses at each stimulator output were separated by an interstimulus interval of 2–3 s. To isolate the coil click from sound originating from the stimulator, the coils were placed inside a soundproof chamber (Model 402.a; Industrial Acoustic Company, USA). To suppress echoes in the recordings, the relevant chamber interior walls were covered with 50 mm thick open-cell foam pads. To convert the digital signal into pascals, the system was calibrated with a 1 kHz, 1 Pa reference sound pressure source (Extech Instruments 407,722, Extech, USA). To allow accurate measurement, we used an omnidirectional pressure microphone (Earthworks M50, Earthworks Audio, USA), which has a flat frequency response (−1.1 to +0.2 dB referenced to our 1 kHz calibration tone) from 3 Hz to 50 kHz and is free of distortion for SPL up to 140 dB(Z). The microphone was connected to a high-input-levelcapacity preamplifier (RNP8380, FMR Audio, USA) and an audio interface with a sample rate of 192 kHz (Behringer U-Phoria UMC404HD, Behringer, Germany).

### Sound processing

First, the TMS electromagnetic artifact was removed by suppressing raw audio from −125 to 500 μs, which is before the acoustic signal from the coil arrives at the microphone, as discussed above. Then the audio was low-pass filtered at 60 kHz to remove high-frequency electronic readout noise and high-pass filtered at 60 Hz to remove low-frequency sounds which mostly originated from the air conditioning. The cut-off frequency for the latter filter was selected post-hoc, as none of the coils produced sound that deviated from the background noise at any frequency below 100 Hz. The high- and low-pass filters were fourth-order Butterworth type applied in both forward and reverse time, resulting in flat frequency response from 80 Hz to 50 kHz (within 1 dB). For these data, we computed the peak SPL [17] in a 0.2 s window around the pulse, the duration of the sound impulse defined as the period for which the sound pressure was within 20 dB of its peak value, and 1/3-octave spectra with the poctave function (Matlab R2018a, Mathworks, USA). The stimulation strength and SPL of the first pulse at a given stimulator output typically differed slightly from the subsequent pulses. This is due to non-idealities, such as hysteresis, in the high-voltage power-supply control in the tested devices. Therefore, all first pulses were excluded from further analysis, but are included in the supplemental dataset. The SPL of subsequent pulses was highly repeatable, having less than 1 dB variation for all coils at ≥20% MSO.

In addition, to estimate the continuous sound level due to rTMS, we synthesized 10 s recordings of rTMS sound as a superposition of individual pulses repeated at 20 Hz. This is the highest repetition frequency in clinical applications using the figure-of-eight type coils that we characterized [18,19].

### Estimation of stimulation strength

To compare the sound between different coils, which provided different stimulation waveforms and MSO, we normalized their stimulation strengths as follows. First, we measured the induced electric field (E-field) corresponding to a depth of 15 mm under the center of the coil in an 85 mm sphere approximating the head [20] using a 70-mm-high triangle-loop probe [21,22]. The E-field was sampled at 100 MHz with an oscilloscope (Tektronix MDO3054, Tektronix, USA). To obtain the effective stimulation strength, the Efield waveform was digitally low-pass filtered with a time constant of 200 μs, corresponding to the approximate strength—duration time constant in primary motor cortex [15,23,24]. The stimulation strength, defined as the absolute peak of the filtered waveform, was then scaled relative to the average resting motor threshold (RMT) by denoting 100% RMT to correspond to 50.3% MSO for the Magstim 70mm Double Coil driven by a Magstim Rapid biphasic stimulator [25]. There is, however, large variability in RMT across individuals. As a representative worst case, we consider a subject whose threshold is the top 5 percentile of 73 patients treated in Ref. [18]. For such a subject the common rTMS setting of 120% RMT corresponds to 167% of our reference average RMT.

### Sound spectrum weighting and exposure limits

For impulsive sounds, most safety standards agree with an instantaneous SPL limit of 140 dB [26], with either C-weighting (e.g. Ref. [27–29]) or Z-weighting (e.g. Ref. [14,30,31]). (For frequencyweighting specifications, see Supplementary Fig. S2.) Of these standards, Directive 2003/10/EC by the European Union (EU) further specifies two lower action values: hearing protection should be provided above 135 dB(C) and must be worn above 137 dB(C) [29]. There are, however, further variations between countries, with some having tighter limits such as 130 dB(Z) in Brazil [26]. Z-weighting (with its flat frequency response) is particularly relevant to the TMS impulsive sound characteristics as it covers both audible and near-ultrasound content [14], whereas Cweighing is defined only up to 20 kHz and therefore limit the sound characterization to the hearing range. With C-weighting, impulsive near-ultrasound content has to be considered separately.

For rTMS, safety limits for continuous sound apply as well. These limits generally use slow A-weighting, are typically (but not always) averaged over 8 h, and vary from 70 to 90 dB(A) across countries (with 85 dB(A) being the most common) [26]. The limits for the referenced standards are summarized in Supplementary Fig. S3. An example of a standard that uses a significantly shorter averaging window is MIL-STD-1474E, which imposes a steady-state noise limit of 85 dB(A) for impulsive noise sources operated for more than 1 s (Limit A) [14]. A less restrictive “Limit B shall be chosen when 85 dB(A) would be equaled or exceeded occasionally or intermittently after all practical design approaches to reduce sound pressure levels to below hazardous levels by engineering principles have been explored” [14]. Limit B has a slope of −3 dB per doubling of exposure duration, ranging from 130 dB(A) for a 0.9 s exposure to 85 dB(A) for an 8 h exposure [14]. The American Conference of Governmental Industrial Hygienists (ACGIH) specifies that noise sources in excess of 80 dB(A) sound level and 3 s duration should be monitored with noise dosimetry [27]. ACGIH follows this with a hard *threshold limit value*, which is identical to MIL-STD-1474E Limit B for total exposure durations greater than 0.9 s [27]; the Australian legislation specifies an identical *exposure standard for noise* [28]. The EU specifies two action values—*lower exposure action value* of 80 dB(A) for providing hearing protection and *upper exposure action value* of 85 dB(A) for mandatory hearing protection together with a list of technical solutions to reduce the exposure—followed by a hard *exposure limit value* of 87 dB(A) [29]. Like the three previously mentioned standards, these EU limits are for an 8 h exposure with a slope of 3 dB per doubling of exposure duration. The US Occupational Safety and Health Administration (OSHA) requires monitoring, including noise measurements and annual audiometric tests, for noise exposure in excess of an *action level* of 85 dB(A) time-weighted average over 8 h. The OSHA *permissible noise exposure* hard limit is 115 dB(A) for durations of less than 15 min and decreases by 5 dB per doubling of duration to 90 dB(A) for exposure of 8 h [30,31]. Finally, there are limits for continuous ultrasound of various durations, ranging from 75 to 110 dB(Z) at 20 kHz to 110–115 dB(Z) at 25 kHz or above [32].

To evaluate the TMS noise in the context of these standards, we computed the peak SPL of single TMS pulses using Z-weighting and C-weighting as well as the sound level of rTMS trains using Aweighting per the international standard on sound level meters (IEC 61672) [17] with splMeter (Audio Toolbox, Matlab R2018a, Mathworks, USA).

## Results

### Coil electric field and stimulation strength

**Fig. 1.**
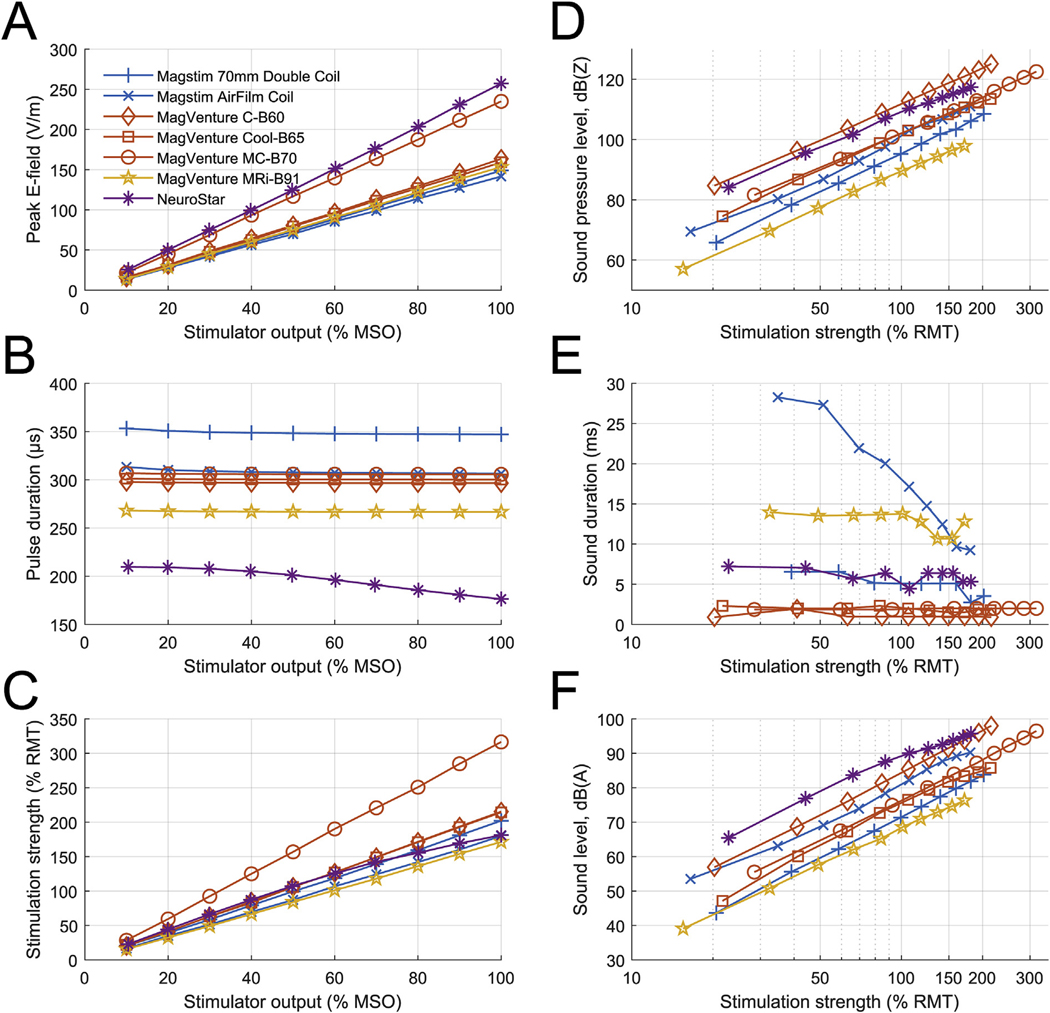
E-field and sound characteristics of various measured TMS coils. (A) Peak E-field value has approximately linear relationship with stimulator output. (B) Pulse durations of air-core coils are approximately constant, whereas pulse duration of NeuroStar iron-core coil starts to drop after about 30% MSO. (C) Stimulation strength estimated with neural model, where 50.3% MSO of Magstim 70mm Double Coil is set to correspond to 100% RMT. (D) Peak sound pressure level (SPL) with Z-weighting, i.e., flat response curve. (E) Duration for which sound is within 20 dB of peak value. (F) Simulated sound level of a 20 Hz rTMS pulse train with A-weighting (slow time weighting).

The peak E-field at 100% MSO varied between 140 and 260 V/m across the tested coils, and it had nearly linear relationship to the stimulator output setting for all coils (Fig. 1A). The pulse durations varied between 176 and 353 μs and were largely stable across the output range of the devices except for the NeuroStar iron-core coil, which had about 19% longer pulse at 10% MSO than at 100% MSO (Fig. 1B). Consequently, for all but the iron-core coil, the devices showed an approximately linear relationship between the simulator capacitor voltage and the estimated stimulation strength (Fig. 1C) (NeuroStar devices, however, have an output calibration feature that can compensate for this nonlinearity).

### Coil sound characteristics

**Fig. 2.**
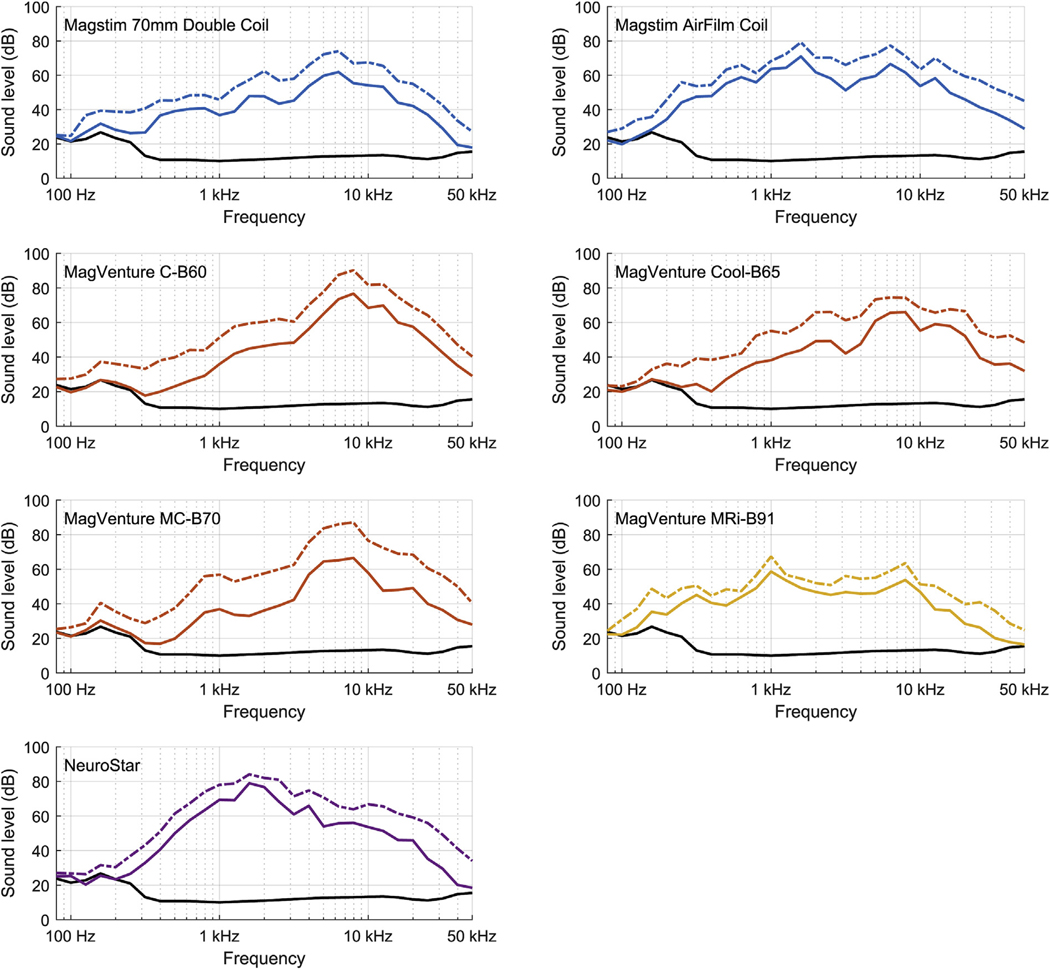
1/3-octave spectra of tested coils at estimated 100% RMT (solid line) and at 100% MSO (dash-dotted line). Solid black line shows ambient sound spectrum. The spectra below 300 Hz are affected by the acoustics of the soundproof chamber (for more detail, see Supplementary material). For all coils, spectra power peak is between 1000 and 10,000 Hz, i.e.,within hearing range (20e20,000 Hz). Spectra at 100% RMT were produced from closest measured stimulator output by assuming that sound waveform is proportional to pulse energy. Due to the 0.2 s time window for each sample, the sound level corresponds to that of a 5 Hz rTMS pulse train.

At a distance of 25 cm, the seven coils had peak SPL of 98–121 dB(Z) at the estimated 167% average RMT (120% individual RMT for a representative high-threshold subject) and 98–125 dB(Z) at 100% MSO (corresponding to 112–135 dB(Z) and 112–139 dB(Z) estimated at a 5 cm distance, respectively) (Fig. 1D). With C-weighting, the peak SPL was 95–118 dB(C) at the estimated 167% average RMT and 96–122 dB(C) at 100% MSO (corresponding to 109–132 dB(C) and 110–136 dB(C) estimated at a 5 cm distance, respectively). With both weightings, MagVenture MRi-B91 was the quietest. There were three distinct click types: very brief and sharp (1–2 ms) from conventional MagVenture coils; medium length (6–7 ms) from NeuroStar and Magstim 70mm Double Coil, and long (>10 ms) from MagVenture MRi-B91 and Magstim AirFilm Coil which have double casing (Fig. 1E). For most coils, the sound duration was comparable across stimulation strengths. For Magstim AirFilm Coil, the click duration decreased from 35 ms to 9 ms when the stimulator output was increased from 20% MSO to 100% MSO. All coils produced broad sound spectra with peak varying between 1 and 8 kHz and spectrum extending up to 30 kHz (Fig. 2). For 5 Hz rTMS at 100% MSO, the 1/3-octave band had sound level between 40 and 69 dB(Z) at 20 kHz and 41–64 dB(Z) at 25 kHz (54–83 dB(Z) and 55–78 dB(Z) at 5 cm). Peak SPL varied approximately proportionally to the stimulation strength squared (Fig. 1D). For example, increasing the stimulation strength by 20% increases the sound level by approximately 3 dB. This is expected from first principles, since the mechanical force causing the sound is a product of the magnetic field and coil current, both of which are proportional to the stimulator capacitor voltage.

**Fig. 3.**
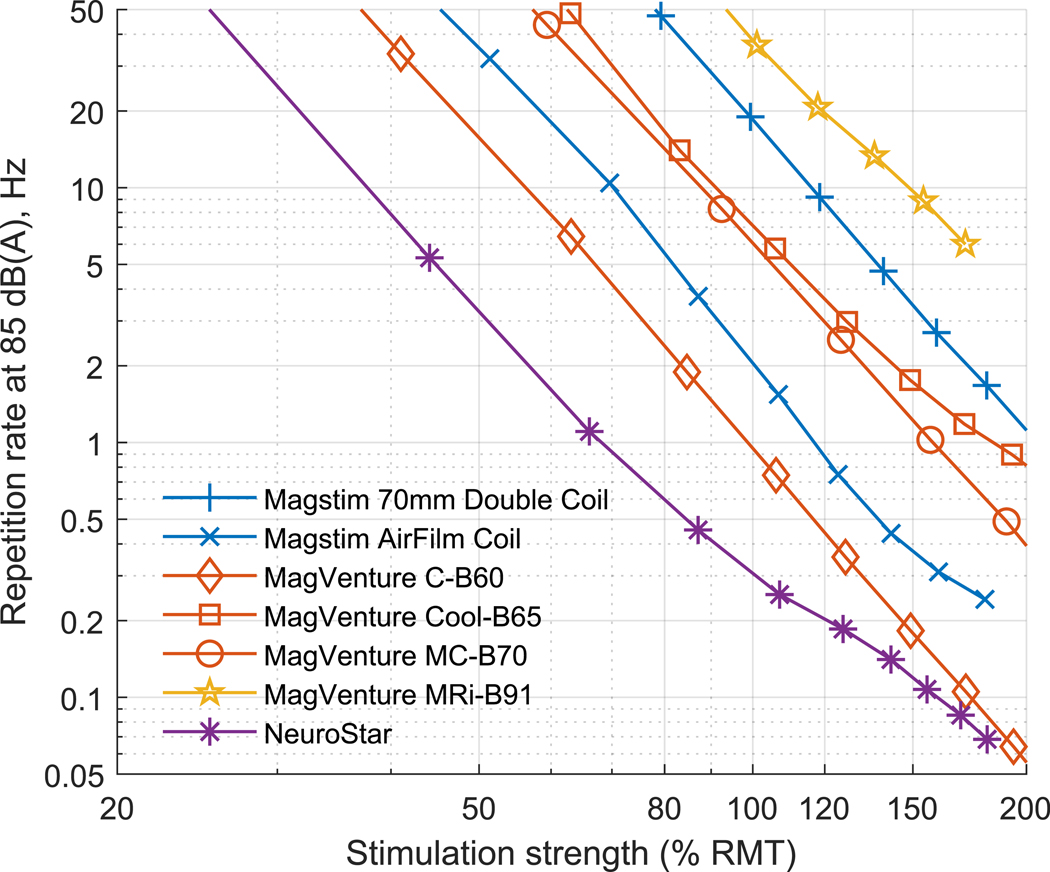
For each tested coil, the rTMS pulse repetition rate corresponding to an estimated airborne sound level of 85 dB(A) for the subject as a function of stimulation strength (in % of average RMT).

The continuous sound for simulated 20 Hz rTMS at 167% average RMT ranged 76–95 dB(A) (90e109 dB(A) at 5 cm), with NeuroStar being the loudest and MagVenture MRi-B91 remaining the quietest but with a much smaller margin than for peak SPL (Fig.1F). At 100% MSO, the sound level would reach 76–98 dB(A) (90–112 dB(A) at 5 cm). Doubling the repetition rate increases the sound level by approximately 3 dB; this scaling is accurate for frequencies up to about 50 Hz, where the sound levels are 4 dB higher than the values shown in Fig. 1F. Doubling the distance from the coil reduces the sound level by 6 dB. Table 2 summarizes how the data presented in Fig. 1 can be scaled approximately to various stimulation strengths, rTMS train frequencies, and coil distances. Fig. 3 uses these scaling laws to compute the rTMS pulse repetition rate at which the subject is exposed to 85 dB(A) as a function of the stimulation strength. For an average subject, the maximum repetition rate for rTMS at 120% RMT varies between 0.2 and 19.9 Hz, and for the top 5 percentile subjects—between 0.1e6.6 Hz.

## Discussion

We measured the far-field airborne sound of TMS coils, at a distance larger than the characteristic dimensions of the coil: Our microphone was placed 25 cm away from the center of the coil, compared to common coil—ear distances of 5 cm for the TMS subject and 30–50 cm for the TMS operator. Nevertheless, we presented simple scaling to estimate the sound level at distances corresponding to the TMS subject or operator. We chose a measurement distance of 25 cm to avoid either sampling the sound spatial distribution or underestimating the SPL due to measuring near a nodal point of a standing wave pattern in the near field [2,15]. This also enabled temporal separation of the sound waveform from the stimulation artifact induced in the microphone hardware, allowing simple suppression of the latter. The main limitations of the present, as well as prior, measurements of TMS sound are that they do not capture the potentially significant nearfield peaks in the SPL or the bone-conducted sound which results from the TMS coil resting against the scalp of the subject [11]. Finally, we did not measure the sound of the TMS coil cable and pulse generator, which could contribute to the sound level, especially for the operator.

Various single-pulse TMS paradigms can reach the maximum device output. In this worst case, the peak SPL of the seven coils ranged 112–139 dB(Z) and 110–136 dB(C) for the subject as well as 98–125 dB(Z) and 96–122 dB(C) for the operator. The Z-weighted values are below the standard-defined limit of 140 dB(Z) for impulsive sound, but for TMS subjects some coils are near this limit. However, the C-weighted peak SPL for MagVenture C–B60 at 100% MSO exceeds the *lower exposure action value* of EU Directive 2003/10/EC [29], which requires that hearing protection be provided for the subjects.

As with the sound of single TMS pulses, there were large differences in the time-averaged steady-state sound levels of pulse trains for rTMS. Even for a subject with an average motor threshold, all coils exceed Limit A of MIL-STD-1474E for the 20 Hz rTMS train at 120% RMT. Further, for an average subject, almost all common rTMS paradigms exceed this limit for most coils (see Fig. 3). For a representative high-threshold subject, a 20 Hz rTMS train at 120% RMT has a sound level of 90–109 dB(A) for the subject and 76–95 dB(A) for the operator. These values would be approximately 3 dB lower for 10 Hz rTMS trains and 4 dB higher for 50 Hz trains, respectively. For the subject, this sound level exceeds Limit A of MIL-STD-1474E for all coils, and for the operator this is true for Magstim AirFilm Coil, MagVenture C–B60, MagVenture MC-B60, and NeuroStar. Further, these coils as well as Magstim 70mm Double Coil and MagVenture Cool-C60 would exceed the ACGIH monitoring threshold for the operator for continuous stimulation trains longer than 3 s (If the distance from the coil is increased to 120 cm, the sound level exposure of the operator would be below the Limit A of MIL-STD-1474E for all tested coils.) For our example high-threshold subject receiving a conventional 10 Hz rTMS depression treatment protocol (75 pulse trains of 4 s duration) [33], the Magstim AirFilm Coil would also exceed the EU *lower exposure action value*, and MagVenture C–B60 and NeuroStar would further exceed Limit B of MIL-STD-1474E, the ACGIH *threshold limit value*, the Australian *exposure standard for noise*, and the EU *upper exposure action value*. Very high repetition rates and intensities, such as 100 Hz at 100% MSO used in magnetic seizure therapy [34,35], may further exceed the US Occupational Safety and Health Administration (OSHA) hard limit of 115 dB(A) for the subject.

In this dataset of commercial TMS systems, all coils had maximum SPL in the hearing range (20–20,000 Hz) (Fig. 2). The sound spectra, however, extended beyond 20,000 Hz, and the 1/3-octave band of near-ultrasound at 20 kHz was between 45 and 72 dB(Z) (59–86 dB(Z) for the subject) for 20 Hz rTMS at 167% average RMT (Fig. 2). These SPLs for the subject exceed the lowest near-ultrasound limit of 75 dB(Z) in the 1/3-octave band at 20 kHz [32] for Magstim AirFilm Coil; MagVenture C–B60, Cool-B65, and MC-B70; and NeuroStar. This underscores the need to measure the TMS sound spectrum to at least 30 kHz, which is much higher than the 8 kHz or 16 kHz bandwidth available in common sound level meters compliant with Class II or Class I of IEC 61672 (even if the meter supports measurement of peak SPL in addition to the timeaveraged steady-state sound level of this standard).

The quietest of the tested coils, MagVenture MRi-B91, is designed for TMS inside a magnetic resonance imaging (MRI) scanner. This result was expected, since the coil is engineered to withstand the increased mechanical forces occurring when TMS coils are exposed to the large static magnetic field in the scanner bore. However, our sound measurements were carried out in the absence of an external magnetic field and therefore cannot be generalized to represent the sound levels inside an MRI scanner, which are expected to be significantly higher.

The previous comparison between TMS devices used sound level meters with a slow time weighting and a measurement bandwidth of only 8 kHz [12], which is insufficient for impulsive sounds [2]. Compared to that study, we observed significantly higher coil SPL and a different order of loudness for the three coils common in the two studies. At 100% MSO and 25 cm distance in our study, Magstim 70mm Double Coil, MagVenture Cool-B65, and NeuroStar registered at 108, 113, and 117 dB(Z), respectively, whereas these coils produced only 90.2, 78.3, and 82.7 dB(A), respectively, in the prior study despite the smaller measurement distance of 10 cm [12]. Specifically, the Magstim and MagVenture coils were respectively 18.3 dB and 35.1 dB louder with the wideb-and width impulse sound measurement at 25 cm, and these numbers rise to 26.2 dB and 43.1 dB after extrapolating the loudness to a matched distance of 10 cm. It should be noted, though, that the version of a particular coil that we tested may have differed from those in other studies; for example, the NeuroStar coil we tested is an early model (version 1.0).

On the other hand, compared to the previous peak SPL measurement using a head phantom [7], we obtained lower SPL for the same coil (Magstim 70mm Double Coil): 105 dB(C) in the acoustic far field versus 127.6 dB(C) in the ear canal in the near field. This difference is only partially explained by the smaller distance between the ear and the coil. The main factor is likely the ear canal model in their head phantom, which mimics the fact that the pressure levels in the ear are higher than the free field pressure levels [36]. The GRAS RA0045 microphone used in those measurements attempts to mimic human ear-canal response curve between 0 and 10,000 Hz (and has a narrow +32 dB resonant peak outside that measurement range at 13.5 kHz, likely amplifying the high-frequency content of the coil click) [37]. In addition, the subject’s or phantom’s ear resides in the acoustic near field of the coil where the emitted sound can form hotspots of increased SPL. All mentioned sound limits, however, are defined for free-field SPL [14,27–31,38] and not for ear canal pressure levels. Further, the free-field airborne SPL limit does not take into account whether the sound source is touching the head, as is the case for a TMS coil, which can lead to sound conducted through the skull bone. Head phantoms, such as the one used in Ref. [5], do not model boneconducted sound either [39].

In summary, we tested a representative sample of different figure-of-eight TMS coils. For both the subject and the operator, the airborne far-field sound from single-pulse TMS remained below exposure limits for all tested coils, with the exception of one coil, which exceeded an exposure action value for the subject at maximum stimulator output. Thus, for single TMS pulses whose strength is near or below the motor threshold, the estimated airborne sound remained generally below exposure limits for the subject, but these estimates do not include additional risks such as near-field hotspot formation or bone-conducted sound. For most tested coils and realistic rTMS scenarios, our measurements indicate that the sound levels reaching the operator holding the TMS coil can exceed some standard exposure limits such as Limit A of MIL-STD-1474E. The sound from rTMS reaching the subject can exceed several standard exposure limits, including MIL-STD-1474E Limit A for all coils. These findings demonstrate the need to use noise mitigation measures such as hearing protection for the subject and increased distance from the coil, hearing protection, and/or noise monitoring for the operator.

In addition to these quantitative observations from our measurements, there are further reasons to be on the side of caution regarding hearing safety of TMS. As discussed above, our and other conventional acoustic measurements of TMS coils do not address the sound conducted through the skull, nor do they account for the possibility of significant spatial peaks of acoustic energy near the coil, both of which could potentially increase the sound pressure in the ear canal. There is indeed evidence suggesting that bone conduction contributes to auditory activation in the brain, since the magnitude of auditory evoked potentials is reduced with a thin block of foam placed between the TMS coil and the subject’s scalp, which may decrease the skull-transmitted sound compared to rigid contact [11]. The sound emitted from the TMS pulse generator and coil cable can also add to the sound level. Further, we characterized only a sample of figure-of-eight TMS coils. Other TMS coils are likely to have similar sound levels, but it is possible that some coils are quieter or louder. Indeed, rTMS with a different kind of coil has been reported to cause permanent hearing loss, presumably due to inappropriately applied earplugs [3]. Finally, the standard limits “should be used as guides in the control of noise exposure and, due to individual susceptibility, should not be regarded as fine lines between safe and dangerous levels” [27], and noise exposure should be reduced below the safety limits, if possible [40]. Indeed, the maximum sound impulses reaching the TMS subject for the majority of coils we tested are comparable with industrial forge hammering (peak SPL 125–133 dB(Z)), where the SPL and number of impulses have been shown to correlate with hearing loss in humans [41]. Moreover, current hearing safety standards only consider hearing loss as an elevated hearing threshold, whereas there is emerging evidence that hearing loss is a more complex phenomenon [42]. For example, degraded inner hair cell synapses were described in animals exposed to loud sounds that do not exhibit any permanent hearing threshold shifts, and such changes are hypothesized to cause suprathreshold hearing deficits in humans [42].

## Conclusion

Commonly used TMS devices have large differences in their sound levels at matched stimulation strength. During rTMS, the airborne sound of some of the tested coils can exceed some standard exposure limits at distances relevant for TMS operators, which implies that exposure should be monitored and mitigated (e.g. with increased distance from the coil or hearing protection). For TMS subjects, in addition to rTMS exceeding some standard exposure limits, single-pulse sound levels can approach exposure limits for stimulation strengths substantially higher than motor threshold. For the subjects there may be additional sources of risk such as skull conduction and near-field sound pressure peaks. These findings and considerations are consistent with the current recommendations of the TMS safety consensus group that “hearing safety concerns for adults be addressed by […] use of approved hearing protection (earplugs or ear muffs)” [4]. The methods and data presented here can inform future refinements of the TMS hearing safety guidelines and the development of quieter TMS devices.

## Supplementary Material

1

## Figures and Tables

**Table 1:** Tested TMS stimulators and coils from Magstim (UK), MagVenture (Denmark), and Neuronetics (USA).

Stimulator	Coil
Magstim Rapid 2 (P/N 3013-US)	70mm Double Coil (P/N 9925–00)^a^ AirFilm Coil (P/N 3910e00)
MagVenture MagPro X100 incl. MagOption (P/N 9016E0731)^b^	CeB60 (P/N 9016E0482) Cool-B65 (P/N 9016E0491) MC-B70 (P/N 9016E0564) MRi-B91 (P/N 9016E0661)
Neuronetics NeuroStar TMS Therapy System (ver. 1.0, P/N 81–00315-000)	NeuroStar (ver. 1.0, P/N 81–00900-000)

a Connected to stimulator with adapter (Magstim, P/N 3110e00).

b “Standard biphasic” pulse mode was used.

**Table 2:** Scaling laws for reported peak SPL and rTMS sound level.

Effect	Change in peak SPL	Change in rTMS sound level
Stimulation strength	$12\text{dB}\cdot \log_{2}\frac{\text{Stim}.\text{strength}[\%\text{RMT}]}{100\%}$	$12\text{dB}\cdot \log_{2}\frac{\text{Stim}.\text{strength}\left[\%\text{RMT}\right]}{100\%}$
Repetition rate	0	$3\text{dB}\cdot \log_{2}\frac{\;\text{Rate}\;[\text{Hz}]}{20\text{Hz}}$
Distance from coil^a^	$-6\text{dB}\cdot \log_{2}\frac{\;\text{Distance}\;[\text{cm}]}{25\text{cm}}$	$-6\text{dB}\cdot \log_{2}\frac{\;\text{Distance}\;[\text{cm}]}{25\text{cm}}$

a Valid down to distance of 5 cm [14].
