# Supplementary material for: Sound comparison of seven TMS coils at matched stimulation strength
Source: Brain Stimul. Author manuscript; Available in PMC 2020 Jun 1. (PMC7263763; doi:10.1016/j.brs.2020.03.004)
Supplement: 1 [file NIHMS1580546-supplement-1.pdf]

Sound comparison of seven TMS coils at matched stimulation strength

Supplementary material

Lari M. Koponen, Stefan M. Goetz, Debara L. Tucci, Angel V. Peterchev

Sound recordings

Based on preliminary measurements, the impulsive sound from TMS pulse generators can be relatively loud. In preliminary measurements with the Tonica MagPro MST stimulator (MagVenture, Denmark), using the same 25 and 100 cm distances from the coil to the microphones as in the final measurement dataset, an impulsive sound source with a peak sound pressure level (SPL) of 104 dB(Z) at 1.7 meters away from the farther microphone was identified. This distance corresponded with the distance to the stimulator. This sound was just 9 dB less than the sound from the tested coil at 1.0 m, i.e., only about 6 dB less if both sound sources would be at equal distances.

To measure accurately the waveform of the coil sound, we had to isolate it from the impulsive sound of the stimulator. We achieved this isolation by measuring the coils inside a soundproof chamber with the TMS pulse generator outside. If one is interested only in the peak SPL and measures from a reasonably close distance to the coil, a soundproof chamber will not be needed. However, in such case, one might want to use at least two microphones (and beamforming) to identify and, if needed, to reject the sound not originating from the direction of the coil. The supplementary sound recording dataset contains a second microphone at 100 cm from the coil, but due to the soundproof chamber the data from this microphone was not needed in the analysis. For the placement of the microphones and the acoustic foam, see Fig. S1B. The purpose of the acoustic foam was to suppress echoes inside the non-anechoic soundproof chamber. We performed a post-hoc analysis to estimate the combined anechoic effect of the chamber walls and the foam by comparing the 1/3-octave spectra of Fig. 2 to an artificially echo-free spectra computed from just the first 10 ms of the sound, with the remaining 140 ms replaced by zeros. The two spectra were within 3 dB between 300 and 30,000 Hz for all coils, and within 1 dB between 400 and 50,000 Hz for all but the two coils with longest sound (Magstim AirFilm Coil and MagVenture MRi-B91). This suggests that echoes and other effects of the finite size of the soundproof chamber had little effect on the measurements above 300 Hz.

The peak SPL was defined as

$$\mathrm{SPL} = \max_{t \in T}\left(94 + 20\log_{10}\frac{|p(t)|}{1\ \mathrm{Pa}}\right)\ \mathrm{dB(Z)} \tag{S1}$$

where T is the 0.2 s time window around a trigger event (from –50 ms to 150 ms) and $p(t)$ is the filtered instantaneous sound pressure [S1]. The ambient noise measured in the soundproof chamber had SPL of 45.0 dB(Z) (95% confidence interval of 43.3–46.6 dB(Z)) with Z-weighting and sound level of 21.2 dB(A) with A-weighting. Consequently, we could measure the pulse durations for pulses down to about 70 dB(Z), i.e., down to 20% MSO for the three quietest coils and down to 10% MSO for the other coils.

As a supplementary material we provide the full set of sound recordings, band-pass filtered from 60 to 60,000 Hz, as described in the methods, and compressed to contain data from –50 ms to 150 ms relative to each pulse, in Matlab file "sound.mat". In this file, the originally 24-bit sound recording data were converted to pascals and stored as 32-bit floating point numbers. In addition to the coil sounds, to assess the background noise level, there are also recordings of 100 events in which no pulse was delivered.

Electric field measurements

The coil E-field was measured with a 70-millimeter-high printed-circuit-board triangular pickup loop with a 50-ohm series resistor, a 3d-printed holder, and a laser-cut alignment tool for mounting the holder on the coils (Fig. S1A). The E-field measurements were estimated to have < 2 mm positional (< 0.05 mm for distance from the bottom of the coil) and < 2° angular uncertainty. With these uncertainties, the field values for each coil have < 2% gain uncertainty. The technical drawings of the measurement probe are provided in file "probe.zip" containing the Gerber files for the two-layer printed-circuit-board probe, an STL file for the 3d-printed probe holder, and an SVG drawing of the laser-cut alignment plate.

The full E-field measurement dataset, including the pulse waveforms, with one pulse per coil per stimulator output, sampled at 100 MHz, is provided in Matlab file "stimulation.mat".

The stimulation strength was computed from the E-field recordings as

$$\Delta V = \max_{t}\left|\int_0^t E(t')\exp\left(-\frac{t-t'}{\tau}\right)\mathrm{d}t'\right|\ , \tag{S2}$$

where ΔV is the stimulation strength, $E(t)$ is the measured E-field waveform, and τ is the strength–duration time constant, here 200 µs.

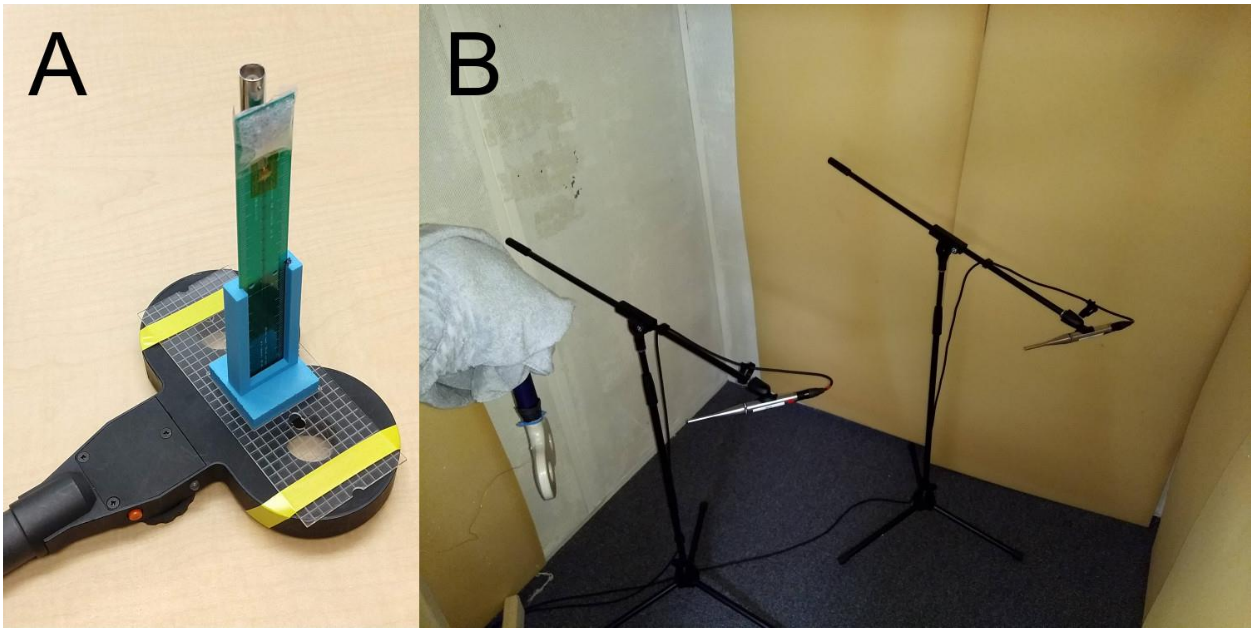

Figure S1. (A) Triangular E-field probe in 3d-printed holder on laser-cut alignment plate positioned on example TMS coil. (B) Sound-recording setup: two microphones were directed at head-side of coil at respective distance of 25 cm and 100 cm measured from microphone capsule. Soundproof chamber provided adequate attenuation of external sound sources including TMS pulse generator; therefore, only data from closer microphone was ultimately needed for analysis. Corners behind coil and microphones were covered with 50 mm thick open-cell foam to suppress echoes. Coil cable was wrapped in towels to suppress its sound.

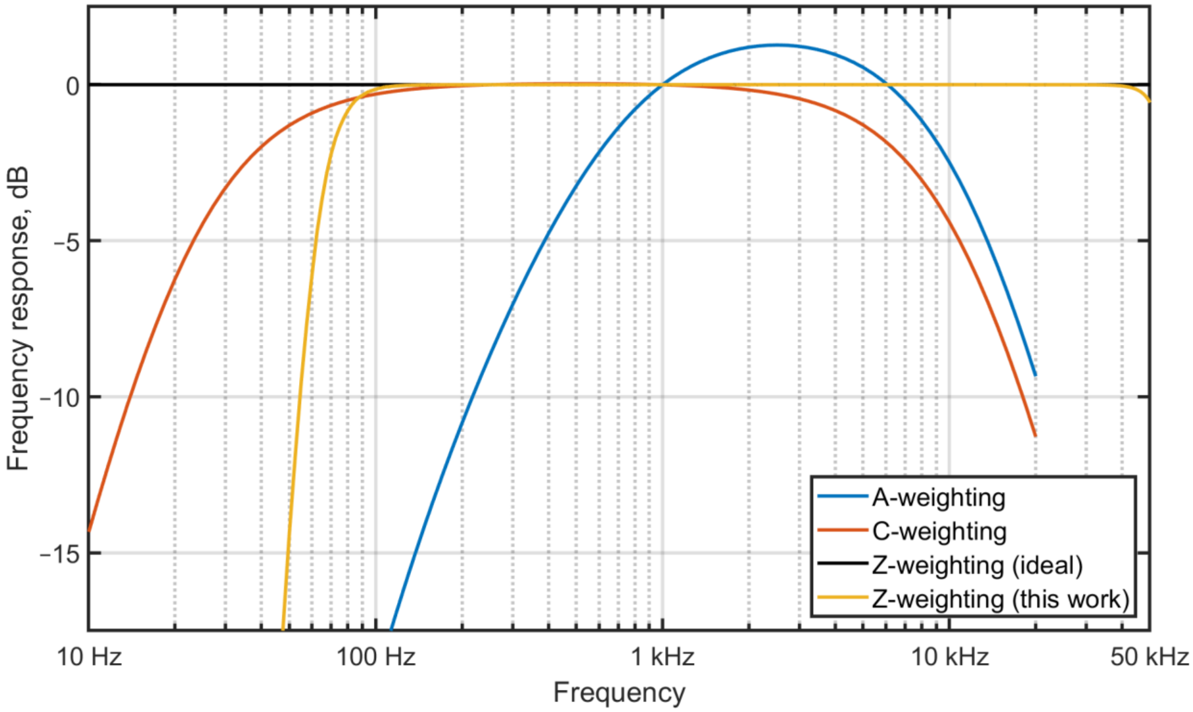

Figure S2. Frequency weightings used in hearing safety standards. A-weighting roughly mimics perceived loudness curve for barely audible sounds, whereas C-weighting mimics that of loud sounds. Ideal Z-weighting corresponds to no weighting at all. In this work, Z-weighting filter had passband from 80 to 50,000 Hz, with sharp low-frequency cut-off selected to reduce noise from non-TMS-coil sources. In practice, a Class II sound-level meter may omit anything above 8 kHz, and a Class I sound-level meter anything above 16 kHz.

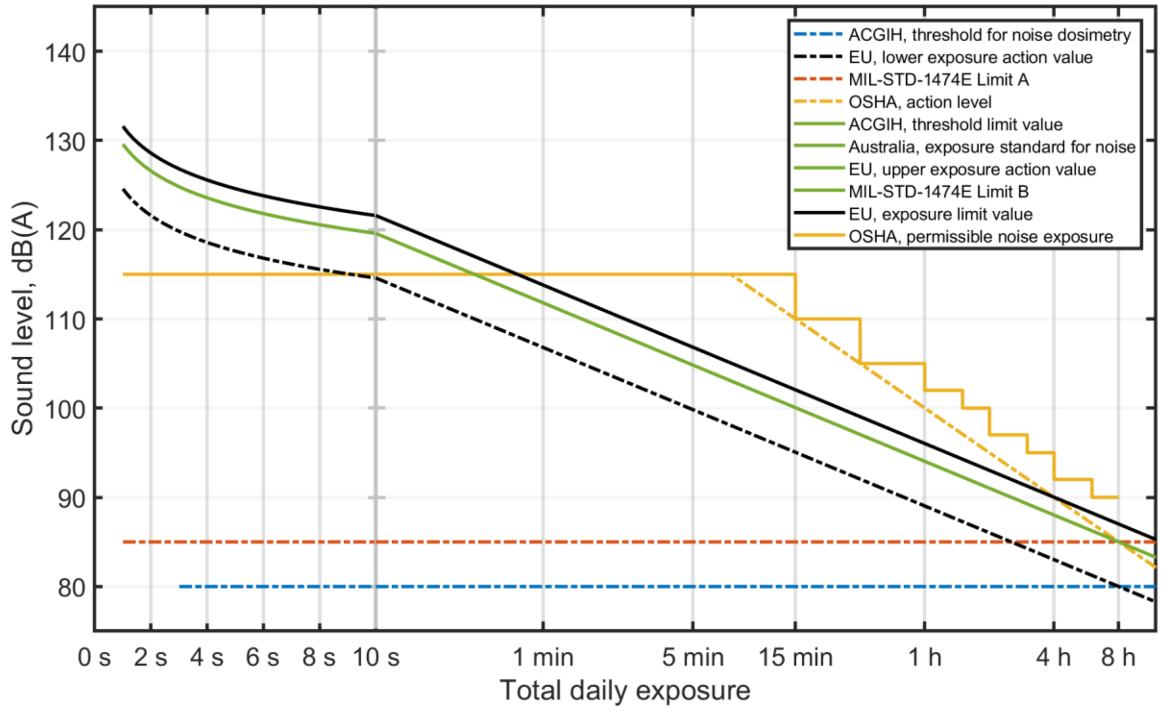

Figure S3. Summary of standard limits on continuous sound exposure from American Conference of Governmental Industrial Hygienists (ACGIH) [S2], US Department of Defense standard MIL-STD-1474E [S3], US Occupational Safety and Health Administration (OSHA) [S4,S5], Australian Government [S6], and the European Union (EU) [S7]. In addition to these continuous sound limits, the standards limit the instantaneous sound pressure level (SPL) respectively to 140 dB(C) [S2], 140 dB(Z) [S3], 140 dB(Z) [S4,S5], 140 dB(C) [S6] and 140 dB(C) [S7]. The EU further defines lower and upper exposure action values for impulsive sounds at 135 and 137 dB(C), respectively. The x-axis is linear up to 10 s and then logarithmic.

References

S1. ANSI/ASA S1.4-2014/Part 1. American National Standard Electroacoustics – Sound Level Meters – Part 1: Specifications (a nationally adopted international standard). Melville, NY: Acoustical Society of America; 2014.

S2. American Conference of Governmental Industrial Hygienists, Threshold Limit Values and Biological Exposure Indices. Cincinnati, OH: ACGIH; 2012.

S3. MIL-STD-1474E. Department of Defense design criteria standard noise limits. Washington, DC: AMSC 9542; 2015.

S4. Occupational Safety and Health Administration, Occupational noise exposure, 29 CFR 1910.95(b)(1) n.d. https://www.govinfo.gov/content/pkg/CFR-2010-title29-vol5/pdf/CFR-2010-title29-vol5-sec1910-95.pdf (accessed March 25, 2019).

S5. Occupational Safety and Health Administration, Occupational noise exposure, 29 CFR 1926.52I n.d. https://www.govinfo.gov/content/pkg/CFR-2014-title29-vol8/pdf/CFR-2014-title29-vol8-sec1926-52.pdf (accessed March 25, 2019).

S6. Work Health and Safety Regulations 2011 n.d. http://www.legislation.gov.au/Details/F2019C00898 (accessed January 22, 2020).

S7. Directive 2003/10/EC of the European Parliament and of the Council of 6 February 2003 on the minimum health and safety requirements regarding the exposure of workers to the risks arising from physical agents (noise) n.d. https://eur-lex.europa.eu/eli/dir/2003/10/2019-07-26 (accessed December 20, 2019).
